# Perfluoroalkyl and Polyfluoroalkyl Substances in Relation to the Participant-Reported Total Pregnancy and Live Birth Numbers among Reproductive-Aged Women in the United States

**DOI:** 10.3390/toxics12080613

**Published:** 2024-08-20

**Authors:** Guangtong Huang, Jiehao Li, Lixin Zhou, Tiantian Duan, Langjing Deng, Pan Yang, Yajie Gong

**Affiliations:** 1 School of Medicine, Jinan University, Guangzhou 510632, China; 18665793625@163.com; 2 Department of Public Health and Preventive Medicine, School of Medicine, Jinan University, Guangzhou 510632, China; a1183930452@outlook.com (J.L.); zlx20010212@icloud.com (L.Z.); 15111872173@163.com (T.D.); dlj7720@stu2021.jnu.edu.cn (L.D.); 3 China Greater Bay Area Research Center of Environmental Health, School of Medicine, Jinan University, Guangzhou 510632, China; 4 School of Environment and Guangdong Key Laboratory of Environmental Pollution and Health, Jinan University, Guangzhou 510632, China; 5 Key Laboratory of Viral Pathogenesis & Infection Prevention and Control (Jinan University), Ministry of Education, Guangzhou 510632, China; 6 School of Public Health, Guangdong Pharmaceutical University, Guangzhou 510006, China

**Keywords:** PFASs, exposure, pregnancy rates, live birth

## Abstract

Perfluoroalkyl and polyfluoroalkyl substances (PFASs), widely utilized in various industries, may pose potential reproductive well-being risks. However, the research on the impact of PFAS exposures on pregnancy and live birth rates remains scarce. To address this gap, we conducted a cross-sectional study using the data from the United States National Health and Nutrition Examination Survey (NHANES) collected between 2013 and 2018. We focused on six PFAS compounds measured in the serum of women aged 20 to 50 years, employing the Poisson regression, Quantile G-composition (Qgcomp), and Weighted Quantile Sum (WQS) regression models. Adjusting for age, racial/ethnic origin, educational level, marital status, family income, body mass index (BMI), menarche age, birth control pill use, and other female hormone consumption, the Poisson regression identified significant negative associations between the individual PFAS exposures and pregnancy and live birth numbers (*p* < 0.05 for all 24 null hypotheses for which the slope of the trend line is zero). The Qgcomp analysis indicated that a one-quartile increase in the mixed PFAS exposures was associated with reductions of 0.09 (95% CI: −0.15, −0.03) in the pregnancy numbers and 0.12 (95% CI: −0.19, −0.05) in the live birth numbers. Similarly, the WQS analysis revealed that a unit increase in the WQS index corresponded to decreases of 0.14 (95% CI: −0.20, −0.07) in the pregnancy numbers and 0.14 (95% CI: −0.21, −0.06) in the live birth numbers. Among the six specific PFAS compounds we studied, perfluorononanoic acid (PFNA) had the most negative association with the pregnancy and live birth numbers. In conclusion, our findings suggest that PFAS exposures are associated with lower pregnancy and live birth numbers among women of reproductive age.

## 1. Introduction

The concerns regarding infertility and adverse pregnancy outcomes are growing. The reports indicate that the global infertility rates among couples of childbearing age range from 12.6% to 17.5%, with an upward trend in risk [1]. Although patients with infertility can achieve pregnancy through in vitro fertilization (IVF) [2], adverse pregnancy outcomes, particularly pregnancy loss, still affect pregnant women [3]. The rise in infertility and pregnancy loss has heightened psychosocial stress in women and imposed significant economic burdens [4,5]. Multiple factors and various potential causes contribute to infertility and pregnancy loss [6,7,8,9,10,11,12,13]. Recent research has emphasized the effects of persistent organic pollutants (POPs) on the reproductive health among women of reproductive age, particularly perfluoroalkyl and polyfluoroalkyl substances (PFASs) [14,15].

PFASs, recognized as synthetic organic compounds characterized by robust carbon–fluorine bonds, exhibit thermal and chemical stability and bioaccumulative properties [16,17,18]. Owing to their nondegradability, some PFASs are known as “forever chemicals” in the environment [15]. Despite the international regulatory measures to limit the PFAS production and consumption in recent decades [15], for example, perfluorooctanesulfonic acid (PFOS) and perfluorooctanoic acid (PFOA) were listed in the Stockholm Convention on Persistent Organic Pollutants (POPs) in 2009 and 2019, respectively [19,20], and certain PFASs, including new replacements similar to PFASs, are still being produced and entering the environment, leading to their increasing detection in human serum [21,22,23]. The research indicates that the majority of the population of the United States has been exposed to certain PFAS compounds [24]. Given the extensive exposure to PFASs, the possible negative reproductive health impacts associated with these chemicals have raised significant concerns.

In experimental studies, exposure to PFASs has been found to impact the reproductive systems of female animals via multiple mechanisms, such as estrogen receptor disruption, interference with steroidogenesis, changes in the endocrine-related gene expression, hindrance of oocyte development, and the induction of premature apoptosis and necrosis in oocytes [25,26,27,28]. Additionally, PFASs might cause adverse placental outcomes through mechanisms involving oxidative stress, disrupted apoptotic signaling, trophoblast dysfunction, and epigenetic alterations [29,30,31,32].

The epidemiologic evidence suggests that exposure to PFASs may be related to increased time to pregnancy, endometriosis-related infertility, and, consequently, infertility [33,34,35,36,37]. Additionally, exposure to PFASs during pregnancy has been found to traverse the placental barrier and pass from pregnant individuals to the fetus [38,39,40,41], affecting the placental DNA methylation [42] and possibly resulting in pregnancy loss [14,43]. However, some research suggested that PFAS exposures might reduce the prevalence of infertility and incidence of pregnancy loss [44,45]. Although studies on the reproductive impacts of PFASs are increasing, the current evidence regarding the detrimental effects of these chemicals on the reproductive well-being of women and the association between PFAS mixtures and infertility and pregnancy loss remain inadequate.

Therefore, we utilized NHANES (2013–2018) data for cross-sectional research to elucidate the connection between PFAS exposures and the participant-reported total pregnancy and live birth numbers in reproductive-aged women, aiming to provide critical and valuable evidence to promote reproductive health.

## 2. Materials and Methods

### 2.1. Research Participants

The NHANES program, managed by the National Center for Health Statistics (NCHS), is a nationwide and comprehensive survey utilizing a sophisticated multi-stage sampling method to assess the nutritional and health conditions of people in the United States. The protocol for NHANES was sanctioned by the NCHS Ethics Review Board, and each participant’s informed consent was secured.

**Figure 1:**
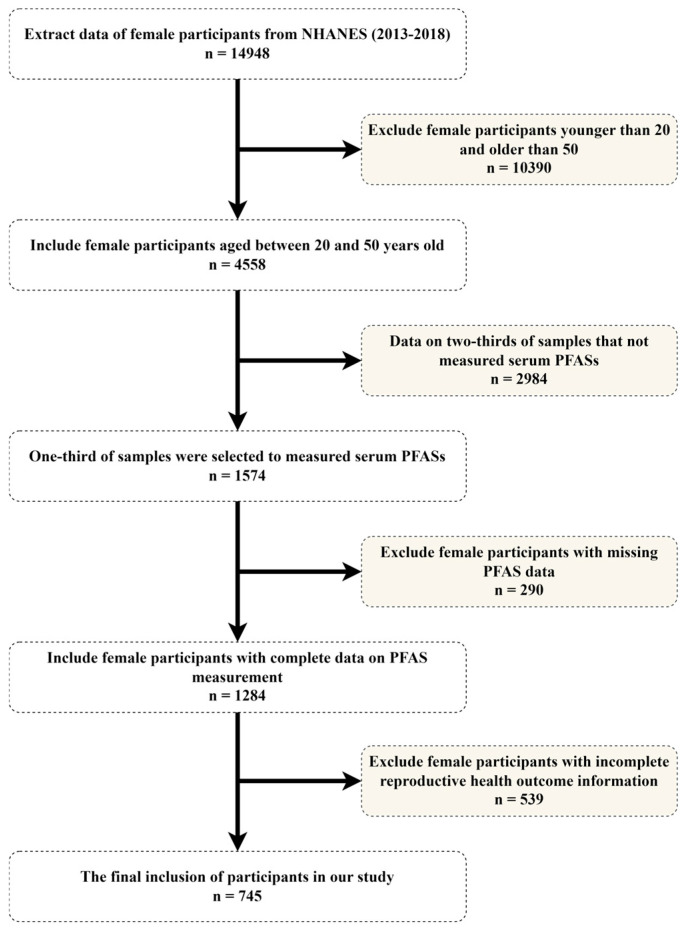
Flowchart illustrating the study’s inclusion and exclusion process.

This research examined data from NHANES for the cycles of 2013–2014, 2015–2016, and 2017–2018, selecting women who matched the study’s inclusion criteria. Figure 1 provided a visual summary of our selection and exclusion protocol. Initially, we enrolled 14,948 women. Subsequently, we excluded 10,390 participants based on age criteria (below 20 or above 50 years old). In the remaining 4558 female participants, NHANES randomly selected one-third of the participants to measure serum PFAS concentrations (1574). Therefore, due to the absence of serum PFAS measurements of the remaining two-thirds of participants, we excluded 2984 participants. Furthermore, we excluded 290 participants from the remaining samples due to missing PFAS data, as well as 539 women for insufficient reproductive health information. Ultimately, the analysis included 745 women, forming the study’s participants.

### 2.2. PFAS Determinations

PFAS data were obtained from the NHANES laboratory database, with all PFASs measured at NHANES mobile examination centers. In each NHANES cycle, a subset consisting of one-third of participants aged ≥ 12 years was chosen to measure PFAS levels in serum. The analytical measurement process was conducted in strict accordance with NHANES quality control and quality assurance guidelines. In brief, NHANES utilized high-performance liquid chromatography–tandem mass spectrometry (HPLC-MS/MS) to detect serum PFASs. Firstly, sample preparation was conducted to remove potential interfering substances, followed by the separation of PFASs using a reverse-phase column. Subsequently, the separated compounds were ionized into charged ions through electrospray ionization and accurately quantified and identified using mass spectrometry analysis. Our study targeted six PFAS compounds, all of which were detected in the three NHANES survey cycles from 2013 to 2018, each with a limit of detection (LOD) of 0.10 μg/L. The six PFAS analytes analyzed were perfluorononanoic acid (PFNA), perfluorodecanoic acid (PFDE), perfluorohexanesulfonic acid (PFHxS), n-perfluorooctanoic acid (n-PFOA), n-perfluorooctanesulfonic acid (n-PFOS), and perfluoromethylheptanesulfonic acid isomers (Sm-PFOS). Consistent with NHANES protocols, values falling beneath the LOD were replaced by the LOD divided by the square root of 2 (LOD/√2). For more detailed information, please refer to the NHANES Survey Content Brochure: https://wwwn.cdc.gov/nchs/data/nhanes/survey-contents-508.pdf (accessed on 10 August 2023).

### 2.3. Reproductive Data

The NHANES employed the Reproductive Health Questionnaire (RHQ) for females aged 12 and older to gather reproductive health data across all survey years with available PFAS data. This dataset was collected by trained researchers using standardized protocols, ensuring consistency across all survey participants. Our analysis focused on the total pregnancy and live birth numbers. Briefly, the participants were asked about the total pregnancy and live birth numbers, birth control pill use, and female hormone consumption. For more detailed information, please refer to the NHANES Questionnaire Data portal: https://wwwn.cdc.gov/nchs/nhanes/search/datapage.aspx?Component=Questionnaire (accessed on 10 August 2023).

### 2.4. Other Variables

Participants reported information on their age, racial/ethnic origin, educational level, marital status, family poverty level index (NHANES variable INDFMMPI, calculated as family monthly income divided by the US Department of Health and Human Services poverty guideline threshold specific to family size, year, and state of residence), body mass index (BMI), and menarche age.

### 2.5. Statistical Analysis

By employing R software (version 4.2.3) for our statistical evaluations, we established the statistical significance threshold at a two-tailed *p*-value of ≤0.05.

Descriptive analyses examined the demographic characteristics and PFAS concentration distributions. We report continuous variables using mean ± SD or median (IQR), and categorical variables are expressed in terms of *n* (%). Given the non-normal distribution of PFAS concentrations, a natural logarithmic transformation was applied to the data for subsequent analyses. Associations between PFAS concentrations across different NHANES survey cycles were evaluated using the Spearman correlation test.

The R package “survey” was utilized to account for the complex multi-stage sampling structure of NHANES. We tested hypotheses of association between individual-level PFAS exposures and pregnancy and live birth numbers using Poisson regression. We converted PFAS measurements to quartiles, represented as independent variables in the regression model by three dummy binary (1 = yes, 0 = no) variables: Q2, Q3, and Q4. Q1 is implied when Q2, Q3, and Q4 are all zero. The dependent variable in each model is the logarithm of the number of pregnancies, or logarithm of number of live births. The exponentiation of the estimated regression coefficient for each quartile variable is the Incidence Ratio (“IR”), which estimates the multiplicative change in number of pregnancies or in number of live births associated with unit change in the quartile variable (i.e., membership in that quartile). The multiplicative change is relative to the reference quartile Q1 in which IR = 1 by definition. For each combination of a specific PFAS exposure and one outcome (number of pregnancies or number of live births), we tested two models controlling for potential confounders: Model 1 included variables representing age, race/ethnicity, education, marital status, family poverty level index, BMI, and menarche age as independent covariates; and Model 2 included all the Model 1 covariates as well as use of birth control pills and use of female hormone supplements.

Using the respective R packages “Qgcomp” and “WQS”, the Quantile G-computation (Qgcomp) and the Weighted Quantile Sum (WQS) regression were used to analyze the influence of PFAS mixtures on the total pregnancy and live birth numbers. On one hand, Qgcomp extends the capabilities of WQS regression, serving as a broader application of the traditional G-computation method, and employs quantized exposures in its regression analysis [46]. Positive and negative weights illustrate how individual components impact the overall effect in both directions on the outcome of interest. On the other hand, the WQS method creates a composite linear index from interrelated predictors, attributing weights according to their relationship with the outcome, summarizing the overall mixture exposures [47]. Before conducting the analysis, the dataset was randomly split into two segments: 40% formed the training set for weight estimation during the ensemble process, with results averaged over 1000 bootstrap samples, and 60% constituted the validation set to estimate the coefficients linked to the mixtures based on these weights. Based on individual model results, each mixture component was constrained to have a negative effect. The β coefficients and 95% confidence intervals (CIs) were reported for both Qgcomp and WQS analyses, where Qgcomp indicated outcomes per quartile increment and WQS demonstrated outcomes per unit increment in its index. In all mixed models, factors such as age, ethnicity, educational background, marital condition, BMI, ratio of family income to poverty, menarche age, contraceptive pill use, and female hormone intake were included in the analysis.

## 3. Results

### 3.1. Demographic Characteristics of Participants

The demographic characteristics of the participants are shown in Table 1. In this study, the participants had an average age (mean ± SD) of 37.1 ± 8.28 years, with non-Hispanic White individuals making up 35.2% of the sample. The mean BMI was 30.5 ± 8.08 kg/m^2^. Approximately 36.8% of the participants had a college degree, 54.1% of the participants were married, and 43.4% had household incomes more than twice the poverty threshold. Regarding the contraceptive practices, 71.0% reported having used birth control pills, while only 4.43% reported using female hormone treatments. For the reproductive health characteristics, the median number of pregnancies was three, and the median number of live births was two.

### 3.2. Distribution and Correlations of PFAS Concentrations

As shown in Table 2, all six PFSA compounds were detectable in the serum of >50% of the participants in all three NHANES cycles during the period 2013–2018. In the combined data from all three cycles, n-PFOS exhibited the highest median concentration, followed in descending order by n-PFOA, Sm-PFOS, PFHxS, PFNA, and PFDE.

**Figure 2:**
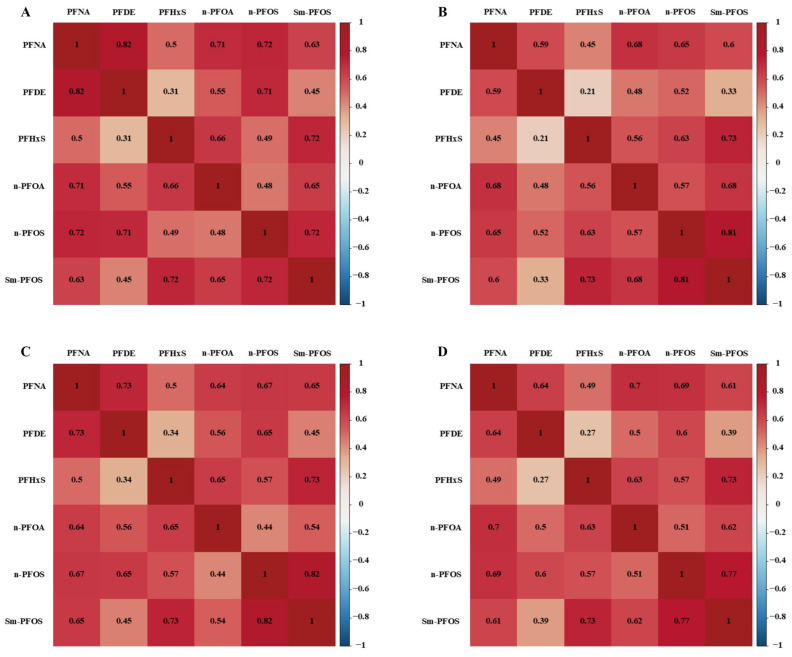
Heat map illustrated the Spearman correlations between six PFASs across different NHANES survey cycles. Note: (**A**) NHANES survey cycle 2013–2014, (**B**) NHANES survey cycle 2015–2016, (**C**) NHANES survey cycle 2017–2018, and (**D**) NHANES survey cycle 2013–2018. Corresponding numeric data are reported in Table S1.

Figure 2 and Table S1 illustrate the Spearman’s correlation coefficients among the six PFAS compounds across the different NHANES survey cycles, showing predominantly low to moderate significantly positive correlations (*r_s_* = 0.27–0.82, *p* values < 0.01).

### 3.3. Association between Individual PFASs and the Total Pregnancy and Live Birth Numbers

**Figure 3:**
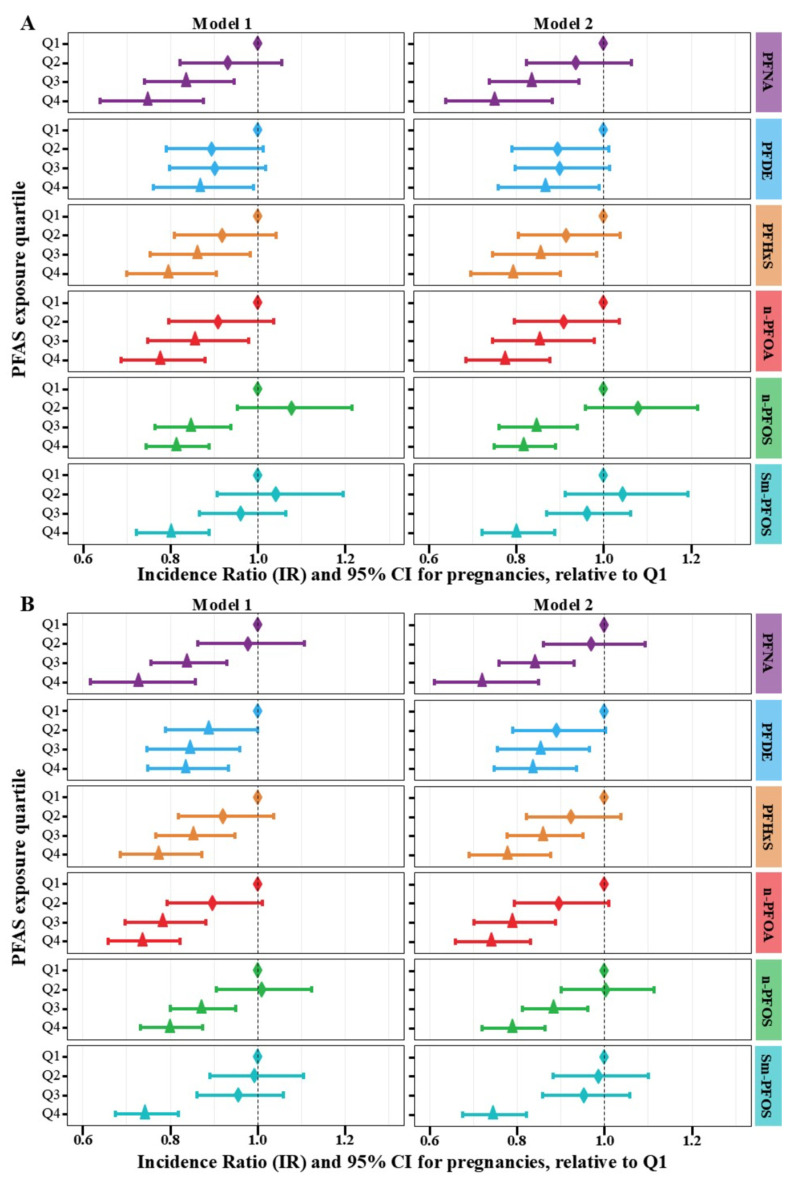
Associations between quantiles of individual PFAS exposure levels and the participant-reported total pregnancy and live birth numbers among 745 women. Note: (**A**) association between PFASs and the number of pregnancies; (**B**) association between PFASs and the number of live births. Cut-offs for PFAS quartiles (Q1, Q2, Q3, and Q4) were determined by the weighted distribution of the whole sample. Model 1 controlled for age, race/ethnicity, education, marital status, body mass index (BMI), ratio of family income to poverty, and menarche age. Model 2 incorporated all covariates in Model 1 along with the use of birth control pills and female hormone intake. Corresponding numeric data are reported in Table S2.

As shown in Figure 3 and Table S2, for all six specific PFAS compounds, and controlling for the two sets of confounders, we observe a consistent pattern of increasing PFAS exposure quartiles associated with decreasing IRs of pregnancies and decreasing IRs of live births (*p* < 0.05 for all 24 null hypotheses for which the slope of the trend line is zero).

### 3.4. Relationship between Exposure to PFAS Mixtures and the Total Pregnancy and Live Birth Numbers

**Figure 4:**
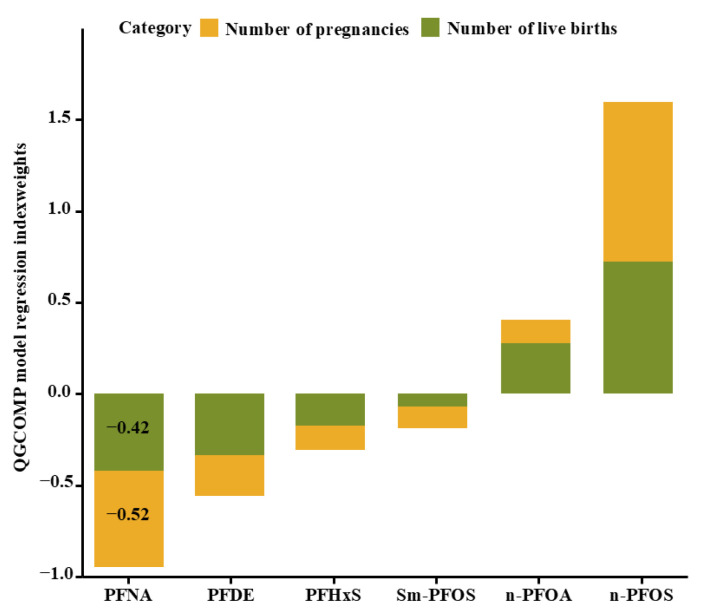
Qgcomp model regression index weights of the PFAS mixtures on the self-reported total pregnancy and live birth numbers. Note: All models incorporated adjustments for age, race/ethnicity, education, marital status, body mass index (BMI), ratio of family income to poverty, menarche age, contraceptive pill use, and female hormone intake. Corresponding numeric data are reported in Table S3.

**Figure 5:**
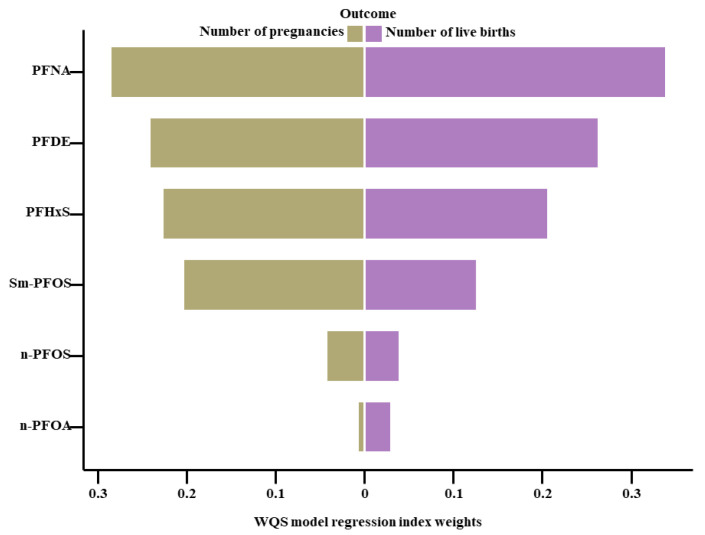
WQS model regression index weights of the PFAS mixtures on the self-reported total pregnancy and live birth numbers. Note: All models incorporated adjustments for age, race/ethnicity, education, marital status, body mass index (BMI), ratio of family income to poverty, menarche age, contraceptive pill use, and female hormone intake. Corresponding numeric data are reported in Table S3.

The mixed impact of PFASs on the total pregnancy and live birth numbers, estimated through the Qgcomp and WQS analyses, was illustrated in Figure 4 and Figure 5 and Table S3. In the Qgcomp, a quartile-level increase in the PFAS mixtures was related to reductions in the pregnancy numbers by 0.09 (95% CI: −0.15, −0.03) and live birth numbers by 0.12 (95% CI: −0.19, −0.05). Among the PFASs with negative weights, PFNA exhibited the most substantial negative impact on the total pregnancy and live birth numbers. In the WQS analysis, an increase of 1 unit in the WQS index of the PFAS mixtures was related to a decrease in the number of pregnancies (0.14, 95% CI: −0.20, −0.07) and decreased live birth numbers (0.14, 95% CI: −0.21, −0.06). Exposure to PFNA was the dominant factor in the estimated mixture effects across all the measured outcomes.

## 4. Discussion

This population-based cross-sectional study revealed a significant inverse relationship between higher PFAS concentrations in the serum and the total pregnancy and live birth numbers among women, as determined by Poisson regression analyses. Additionally, higher PFAS mixture concentrations were significantly linked to fewer pregnancies and live births in the Qgcomp and WQS models, with PFNA being identified as the primary contributor. These findings suggest that higher concentrations of PFASs may adversely impact the reproductive health of women of childbearing age in the United States.

We compared the median PFAS concentrations in the serum with those reported in the NHANES and similar studies globally. Consistent with the earlier research, a significant decrease in the PFAS concentrations was observed across the successive NHANES survey cycles [48,49], with the median concentration level of n-PFOS being the highest [23,39]. We found in this study that the highest median level of n-PFOS (1.90 ng/mL) across all the NHANES survey cycles was lower than in previous studies conducted in Ma’anshan during three trimesters of pregnancy (5.10–5.44 ng/mL) [50] and Massachusetts (2.4 ng/mL) [51] but exceeded the levels of the pregnant women from Tangshan (1.21 ng/mL) [42] and Henan (0.35 ng/mL) (0.35 ng/mL) [52], China. PFOS may be permitted or specifically exempted in certain industries [19], resulting in a median concentration of PFOS in the whole blood exceeding 1 ng/mL. The regional disparities in the PFAS exposure patterns may be attributed to variations in the economic conditions, dietary habits, lifestyles, and the use of consumer products.

Similar to the previous studies, research conducted in Singapore [53] observed that the plasma PFOS and PFDE concentrations were inversely correlated with the fecundability of women and clinical pregnancy, and the PFAS mixtures were inversely related to the fecundability, clinical pregnancy, and live birth outcomes, with PFDE being the most significant contributor. However, in their research, they did not observe any relationship between the individual PFASs and live birth and any evidence of an association between the fertility outcomes and PFHxS and PFNA. The study conducted by Lum et al. found that PFOA and PFNA might be related to a reduced probability of pregnancy [54]. We observed significant negative correlations between the six PFASs and the total pregnancy and live birth numbers in our study. The results were similar to a study in Sweden [55], which found that the PFOA serum levels in early pregnancy had an adverse impact on sporadic pregnancy loss, but it did not observe any association with other PFASs. Our results were inconsistent with a study using the data from the NHANES (2013–2016) [44], observing that low PFAS concentrations might be associated with a reduction in infertility. Similarly, a study conducted in Beijing observed that the majority of the PFASs did not correlate with early pregnancy loss [45]. The variations in the outcomes could be attributed to disparities in the PFAS exposure concentrations, regional variations, and methodological differences in the study design, PFAS assessment, and analytical methods.

The effects of PFASs on women’s fecundability have been explored in numerous studies. PFASs may disrupt estrogen synthesis and metabolism, causing abnormal hormone levels, damaging reproductive cells to affect their development and function, and ultimately resulting in infertility [25,56,57,58]. Additionally, PFASs can impair the gap junction intercellular communication in cumulus cells–oocyte complexes and generate reactive oxygen species in the fetal ovary, causing apoptosis and necrosis in mammalian oocytes [27,59]. Previous studies have indicated that PFASs may increase the infant mortality rate and decrease the probability of live births [60,61]. However, the mechanisms underlying the impacts of PFAS exposures on pregnancy loss remain unknown and may involve abnormal placentation. PFASs might induce apoptosis in human placental syncytiotrophoblasts, disrupt placental endocrine function, and affect placental development [62,63]. Although the focuses of these studies vary, they unanimously suggest that the adverse effects experienced by the female reproductive system may eventually result in infertility and pregnancy loss.

This cross-sectional study utilizes a large female sample from the NHANES to investigate, for the first time, the relationship between PFAS exposures and self-reported total pregnancy and live birth numbers, providing relatively comprehensive epidemiological evidence on the adverse effects of PFASs on reproductive outcomes. Additionally, the use of three distinct models for the evaluation of the individual and combined effects of the PFASs on the total pregnancy and live birth numbers is a notable advantage of our study. Although these analytical models may not be novel and have been widely used, the comprehensive and integrated application of these models has strengthened the reliability of our findings.

However, some limitations must be acknowledged. The cross-sectional design was the main limitation, making it impossible to identify the causal connection between exposure to PFASs and the total pregnancy and live birth numbers. Furthermore, using serum PFAS concentrations at a single time point to estimate women’s exposure levels may have led to bias. Despite adjusting for multiple confounders, the influence of unmeasured or unknown confounders cannot be completely ruled out. Overall, more prospective research is needed in the future to explore the actual impacts of PFAS exposures on the reproductive well-being of women.

## 5. Conclusions

In conclusion, our study demonstrated that PFAS exposures are associated with a decrease in the total pregnancy and live birth numbers in women. For PFAS mixtures, PFNA was identified as the major negative factor. Our findings may serve as a foundation for further research to corroborate our findings.

## Figures and Tables

**Table 1:** Baseline characteristics of 745 women, NHANES 2013–2018 (*n* = 745).

Characteristics	
Age (years), mean ± SD	37.1 ± 8.28
BMI (kg/m^2^), mean ± SD	30.5 ± 8.08
Menarche age (years), mean ± SD	12.5 ± 1.79
Race/Ethnic origin, *n* (%)	
Mexican American	131 (17.6)
Other Hispanic	66 (8.86)
Non-Hispanic White	262 (35.2)
Non-Hispanic Black	165 (22.1)
Others	121 (16.2)
Education, *n* (%)	
Less than middle school	45 (6.04)
Middle school	108 (14.5)
High school graduate	152 (20.4)
College degree	274 (36.8)
College graduate or above	166 (22.3)
Marital status, *n* (%)	
Married	403 (54.1)
Widowed	12 (1.61)
Divorced	72 (9.66)
Separated	52 (6.98)
Never married	126 (16.9)
Living with partner	80 (10.7)
Ratio of family income to poverty, *n* (%)	
<1	222 (29.8)
1–2	200 (26.8)
>2	323 (43.4)
Ever taken birth control pills, *n* (%)	
Yes	529 (71.0)
No	216 (29.0)
Ever use female hormones, *n* (%)	
Yes	33 (4.43)
No	712 (95.6)
Total pregnancy numbers (*n*), median (IQR)	3 (2, 4)
Total live birth numbers (*n*), median (IQR)	2 (1, 3)

**Table 2:** Describes the distribution of PFAS exposure concentrations, NHANES 2013–2018 (*n* = 745).

PFASs	LOD, μg/L	Cycle 2013–2014, *n* = 256	Cycle 2015–2016, *n* = 271	Cycle 2017–2018, *n* = 218	Cycles 2013–2018, *n* = 745
PFNA	0.10				
*n* > LOD		252 (98.44%)	264 (97.42%)	184 (84.40%)	700 (93.96%)
Median (IOR)		0.50 (0.30, 0.70)	0.40 (0.20, 0.70)	0.30 (0.15, 0.50)	0.40 (0.20, 0.70)
PFDE	0.10				
*n* > LOD		179 (69.92%)	142 (52.40%)	176 (80.73%)	497 (66.71%)
Median (IOR)		0.20 (0.07, 0.30)	0.10 (0.07, 0.20)	0.20 (0.10, 0.30)	0.10 (0.07, 0.20)
PFHxS	0.10				
*n* > LOD		250 (97.66%)	262 (96.68%)	216 (99.08%)	728 (97.72%)
Median (IOR)		0.60 (0.40, 1.00)	0.50 (0.30, 1.00)	0.50 (0.30, 0.80)	0.50 (0.30, 0.90)
n-PFOA	0.10				
*n* > LOD		254 (99.73%)	267 (98.52%)	218 (100%)	739 (99.19%)
Median (IOR)		1.20 (0.70, 1.80)	0.80 (0.50, 1.40)	0.70 (0.50, 1.20)	0.90 (0.60, 1.40)
n-PFOS	0.10				
*n* > LOD		254 (99.73%)	268 (98.89%)	218 (100%)	740 (99.33%)
Median (IOR)		2.30 (1.30, 3.60)	1.80 (1.20, 2.92)	1.60 (1.00, 2.60)	1.90 (1.20, 3.00)
Sm-PFOS	0.10				
*n* > LOD		250 (97.66%)	264 (97.42%)	217 (99.54%)	731 (98.12%)
Median (IOR)		0.60 (0.40, 1.10)	0.60 (0.40, 1.10)	0.60 (0.40, 0.90)	0.60 (0.40, 1.00)

Note: IOR, interquartile range.

## Data Availability

The data of this study are publicly available from the United States National Health and Nutrition Examination Survey (NHANES) official website: https://www.cdc.gov/nchs/nhanes/ (accessed on 27 July 2023).

## Supplementary Materials

The following supporting information can be downloaded at https://www.mdpi.com/article/10.3390/toxics12080613/s1, Table S1: Spearman correlation of PFAS exposure levels during different NHANES survey cycles (2013–2014, 2015–2016, 2017–2018, and 2013–2018); Table S2: Relative proportion (RP) of participant-reported total pregnancy and live birth numbers by PFAS exposure levels; Table S3: Associations of mixed PFAS exposures and the total pregnancy and live birth numbers in 745 women.
