# Supplementary material for: Perfluoroalkyl and Polyfluoroalkyl Substances in Relation to the Participant-Reported Total Pregnancy and Live Birth Numbers among Reproductive-Aged Women in the United States
Source: Toxics. 2024 Aug 20;12(8):613. doi: 10.3390/toxics12080613 (PMC11359323; doi:10.3390/toxics12080613)
Supplement: Supplementary file 1 [file toxics-12-00613-s001.zip › toxics-3129489-supplementary.pdf]

Supplemental Materials

Perfluoroalkyl and Polyfluoroalkyl Substances in Relation to the Participant-Reported Total Pregnancy and Live Birth Numbers among Reproductive-Aged Women in the United States

**Tables of Contents**		**Page**
**Table S1**	Spearman correlation of PFAS exposure levels during different NHANES survey cycles (2013–2014, 2015–2016, 2017–2018, and 2013–2018).	2
**Table S2**	Incidence Ratio (IR) of participant-reported total pregnancy and live birth numbers by PFAS exposure Quartile.	4
**Table S3**	Associations of mixed PFAS exposures and the total pregnancy and live birth numbers in 745 women.	6

Table S1. Spearman correlation of PFAS exposure levels during different NHANES survey cycles (2013–2014, 2015–2016, 2017–2018, and 2013–2018).

Variable	PFNA	PFDE	PFHxS	n-PFOA	n-PFOA	Sm-PFOS
NHANES survey cycle 2013–2014						
PFNA	1.00					
PFDE	0.82	1.00				
PFHxS	0.50	0.31	1.00			
n-PFOA	0.71	0.55	0.66	1.00		
n-PFOS	0.72	0.71	0.49	0.48	1.00	
Sm-PFOS	0.63	0.45	0.72	0.65	0.72	1.00
NHANES survey cycle 2015–2016						
PFNA	1.00					
PFDE	0.59	1.00				
PFHxS	0.45	0.21	1.00			
n-PFOA	0.68	0.48	0.56	1.00		
n-PFOS	0.65	0.52	0.63	0.57	1.00	
Sm-PFOS	0.60	0.33	0.73	0.68	0.81	1.00
NHANES survey cycle 2017–2018						
PFNA	1.00					
PFDE	0.73	1.00				
PFHxS	0.50	0.34	1.00			
n-PFOA	0.64	0.56	0.65	1.00		
n-PFOS	0.67	0.65	0.57	0.44	1.00	
Sm-PFOS	0.65	0.45	0.73	0.54	0.82	1.00
NHANES survey cycles 2013–2018						
PFNA	1.00					

PFDE	0.64	1.00				
PFHxS	0.49	0.27	1.00			
n-PFOA	0.70	0.50	0.63	1.00		
n-PFOS	0.69	0.60	0.57	0.51	1.00	
Sm-PFOS	0.61	0.39	0.73	0.62	0.77	1.00

Table S2. Incidence Ratio (IR) of participant-reported total pregnancy and live birth numbers by PFAS exposure Quartile.

Exposure	IR of pregnancies		IR of live births	
	Model 1	Model 2	Model 1	Model 2
PFNA				
Q1	1 (Reference)	1 (Reference)	1 (Reference)	1 (Reference)
Q2	0.94 (0.83, 1.06)	0.93 (0.82, 1.06)	0.98 (0.86, 1.11)	0.97 (0.86, 1.09)
Q3	**0.84 (0.74, 0.94)** **	**0.84 (0.74, 0.95)** **	**0.84 (0.76, 0.93)** **	**0.84 (0.76, 0.93)** **
Q4	**0.75 (0.64, 0.88)** **	**0.75 (0.64, 0.88)** ***	**0.73 (0.62, 0.86)** ***	**0.72 (0.61, 0.85)** ***
p for trend	< 0.001	< 0.001	< 0.001	< 0.001
PFDE				
Q1	1 (Reference)	1 (Reference)	1 (Reference)	1 (Reference)
Q2	0.90 (0.79, 1.01)	0.90 (0.79, 1.01)	**0.89 (0.79, 1.00)** *	0.89 (0.79, 1.00)
Q3	0.90 (0.80, 1.02)	0.90 (0.80, 1.02)	**0.85 (0.75, 0.96)** *	**0.85 (0.76, 0.97)** *
Q4	**0.87 (0.76, 0.99)** *	**0.87 (0.76, 0.99)** *	**0.84 (0.75, 0.93)** **	**0.84 (0.75, 0.94)** **
p for trend	0.021	0.023	0.002	0.003
PFHxS				
Q1	1 (Reference)	1 (Reference)	1 (Reference)	1 (Reference)
Q2	0.91 (0.81, 1.04)	0.92 (0.81, 1.04)	0.92 (0.82, 1.04)	0.92 (0.82, 1.04)
Q3	**0.86 (0.75, 0.98)** *	**0.86 (0.75, 0.98)** *	**0.85 (0.77, 0.95)** **	**0.86 (0.78, 0.95)** **
Q4	**0.79 (0.70, 0.90)** **	**0.80 (0.70, 0.91)** **	**0.78 (0.69, 0.87)** ***	**0.78 (0.69, 0.88)** ***
p for trend	< 0.001	< 0.001	< 0.001	< 0.001
n-PFOA				
Q1	1 (Reference)	1 (Reference)	1 (Reference)	1 (Reference)
Q2	0.91 (0.80, 1.04)	0.91 (0.80, 1.04)	0.90 (0.79, 1.01)	0.90 (0.79, 1.01)
Q3	**0.85 (0.75, 0.98)** *	**0.86 (0.75, 0.98)** *	**0.78 (0.70, 0.88)** ***	**0.79 (0.70, 0.89)** ***
Q4	**0.78 (0.69, 0.88)** ***	**0.78 (0.69, 0.88)** ***	**0.74 (0.66, 0.82)** ***	**0.74 (0.66, 0.83)** ***

p for trend	< 0.001	< 0.001	< 0.001	< 0.001
n-PFOS				
Q1	1 (Reference)	1 (Reference)	1 (Reference)	1 (Reference)
Q2	1.08 (0.96, 1.22)	1.08 (0.95, 1.22)	1.01 (0.91, 1.12)	1.00 (0.90, 1.12)
Q3	**0.85 (0.76, 0.94)** **	**0.85 (0.76, 0.94)** **	**0.87 (0.80, 0.95)** **	**0.88 (0.81, 0.96)** **
Q4	**0.82 (0.75, 0.89)** ***	**0.81 (0.75, 0.89)** ***	**0.80 (0.73, 0.87)** ***	**0.79 (0.72, 0.87)** ***
p for trend	< 0.001	< 0.001	< 0.001	< 0.001
Sm-PFOS				
Q1	1 (Reference)	1 (Reference)	1 (Reference)	1 (Reference)
Q2	1.04 (0.91, 1.19)	1.04 (0.91, 1.20)	0.99 (0.89, 1.11)	0.99 (0.88, 1.10)
Q3	0.96 (0.87, 1.06)	0.96 (0.87, 1.07)	0.96 (0.86, 1.06)	0.95 (0.86, 1.06)
Q4	**0.80 (0.72, 0.89)** ***	**0.80 (0.72, 0.89)** ***	**0.74 (0.68, 0.82)** ***	**0.75 (0.68, 0.82)** ***
p for trend	< 0.001	< 0.001	< 0.001	< 0.001

Note:

Model 1 controlled for age, race/ethnicity, education, marital condition, BMI, ratio of family income to poverty, and menarche age.

Model 2 incorporated all covariates in Model 1 along with the usage of birth control pills and female hormone intake.

Values in bold indicate statistically significant results ($p < 0.05$).

* $p < 0.05$; ** $p < 0.01$; *** $p < 0.001$.

Table S3. Associations of mixed PFAS exposures and the total pregnancy and live birth numbers.

Outcome	Number of pregnancies		Number of live births	
	β (95% CI)	Mixture Weights	β (95% CI)	Mixture Weights
Qgcomp	**-0.09 (-0.15, -0.03)** **		**-0.12 (-0.19, -0.05)** ***	
PFNA		-0.52		-0.42
PFDE		-0.22		-0.34
PFHxS		-0.13		-0.17
n-PFOA		0.13		0.28
n-PFOS		0.87		0.72
Sm-PFOS		-0.12		-0.07
WOS regression	**-0.14 (-0.20, -0.07)** ***		**-0.14 (-0.21, -0.06)** ***	
PFNA		0.34		0.28
PFDE		0.26		0.24
PFHxS		0.21		0.23
n-PFOA		0.13		0.20
n-PFOS		0.04		0.04
Sm-PFOS		0.03		0.01

Note:
In WQS regression, we constrained each mixture component effect to be negative based on findings from the individual model.
All models incorporated adjustments for age, race/ethnicity, education, marital status, BMI, ratio of family income to poverty, menarche age, contraceptive pill usage, and female hormone intake.
Values in bold indicate statistically significant results ($p < 0.05$).
* $p < 0.05$; ** $p < 0.01$; *** $p < 0.001$.
